# Nomogram model for prognosis of early colorectal cancer after endoscopic therapy: integration of intestinal microbiota and clinicopathological parameters

**DOI:** 10.3389/fonc.2025.1559045

**Published:** 2025-09-23

**Authors:** Yanli Zhu, Fang Yang, Lijun Meng, Xiaoling Zhang, Yongsheng Chang, Lanfang Zhang

**Affiliations:** Department of Gastroenterology, The First Affiliated Hospital of Henan Medical University, Xinxiang, China

**Keywords:** early colorectal cancer, intestinal flora, clinical pathological parameters, prognosis of endoscopic therapy, nomogram model

## Abstract

**Objective:**

To integrate the characteristics of intestinal flora and clinical pathological parameters in patients with early colorectal cancer, and to construct and validate a nomogram model for predicting the prognosis of endoscopic therapy.

**Methods:**

The data of 80 patients with early colorectal cancer receiving endoscopic treatment from January 2019 to June 2024 were retrospectively collected. They were randomly divided into a training set (n = 56) and a validation set (n = 24) at a ratio of 7:3. The factors related to prognosis were screened by univariate analysis and multivariate Logistic regression analysis regression, so as to construct Nomogram model, calculate C-index, and draw the calibration curve. The clinical application value of the model was evaluated using decision curve analysis (DCA).

**Results:**

There was no significant difference in the incidence of poor prognosis, intestinal flora, and clinical pathological parameters between the training set and the validation set (all *P*>0.05). Multivariate Logistic regression analysis showed that tumor diameter, Shannon-Wiener index, relative abundance of Fusobacterium nucleatum, relative abundance of Bacteroides fragilis, relative abundance of Bifidobacterium, and relative abundance of Lactobacilli were the independent influencing factors for poor prognosis of endoscopic therapy (all *P* < 0.05). The nomogram prediction model was further constructed, and the nomogram model had good calibration and fitting between prediction and reality in the training set and the validation set. ROC curves were shown in the training set and the validation set; AUC of the nomogram model for predicting the prognosis of endoscopic therapy was 0.979(95% *CI*: 0. 946-1.000) and 0. 821(95% *CI*: 0.516-1.000).

**Conclusion:**

Nomogram model based on intestinal flora and clinical pathological parameters can effectively predict the prognosis of early colorectal cancer patients after endoscopic treatment with good accuracy and reliability, which is expected to provide an important reference for the development of clinical individualized treatment plan and guide the accurate treatment and management of patients.

## 1 Introduction

Colorectal cancer is a common malignant tumor in the digestive tract, including colon cancer and rectal cancer. Its incidence is closely related to lifestyle and genetic factors. Patients often present with changes in bowel habits, hematochezia, abdominal pain and other symptoms (1–3). The early detection and treatment of colorectal cancer has a relatively good prognosis, while in the late stage, metastasis may occur, making the treatment more difficult (4, 5). The intestinal flora plays an important role in the occurrence and development of colorectal cancer (6). The changes in the composition and function of intestinal microbial communities may be intertwined with the evolution of colorectal cancer by regulating the immune response, affecting metabolic pathways and other mechanisms (7–9). For patients with early colorectal cancer, the specific characteristics of the intestinal flora is likely to be one of the key factors affecting the prognosis, but this research has not yet been fully carried out, and the synergistic mechanism between the intestinal flora and traditional clinical pathological parameters needs to be further clarified. Clinical pathological parameters, such as tumor differentiation and infiltration depth, have always been an important basis for evaluating the prognosis of tumor patients, but they have certain limitations in reflecting individual differences of patients, making it difficult to predict the prognosis comprehensively and accurately (10). Therefore, it is of great clinical significance to construct a more perfect prognosis prediction model by organically combining the emerging factor of intestinal flora with traditional clinical pathological parameters. Based on this, the purpose of this study was to construct Nomogram model using scientific statistical methods through systematic collection and analysis of data, and strict validation, in order to provide a more accurate and reliable tool for the prognosis evaluation of endoscopic treatment of early colorectal cancer patients, and promote the clinical diagnosis and treatment decisions to a more precise and individual direction.

## 2 Data and methods

### 2.1 Study subjects

Patients with early colorectal cancer who received endoscopic treatment between January 2019 and June 2024 were included retrospectively with approval by the Ethics Committee. According to the inclusion and exclusion criteria, 80 patients, who were divided into a training set (n = 56) and a validation set (n = 24) at a ratio of 7:3 using the random number table method, were included. All patients signed informed consent forms.

### 2.2 Inclusion and exclusion criteria

#### 2.2.1 Inclusion criteria

(1) Early colorectal cancer confirmed by pathology. (2) Endoscopic treatment is performed in hospital, and the operation records are complete, including information on the operation method and resection range. (3) Complete clinical and pathological data can be provided, including details of the patient’s age, gender, tumor location, size, gross morphology, differentiation degree, and presence of lymphovascular invasion. (4) Intestinal flora is detected before treatment. The detection method meets the standards and specifications, and there is detailed flora detection data, including flora diversity index, the relative abundance of specific flora, etc. (5) The patient signed the informed consent form, agreed to use its clinical data in this study, and cooperated with the follow-up investigation.

#### 2.2.2 Exclusion criteria

(1) Combined with other medical history of malignant tumor (except for non-invasive cancers such as cured basal cell carcinoma of skin and carcinoma *in situ* of cervix). (2) There are serious systemic diseases. (3) Other anti-tumor treatments such as chemoradiotherapy and targeted therapy have been received before this endoscopic treatment. (4) There are serious organic lesions in the intestine (such as intestinal obstruction, intestinal perforation, and severe active stage of inflammatory bowel disease). (5) Patients with mental or cognitive dysfunction cannot cooperate to complete the research procedures such as intestinal flora detection, clinical data collection and follow-up investigation.

### 2.3 Treatment methods

#### 2.3.1 Endoscopic mucosal resection

Patients need to complete bowel preparation to ensure intestinal cleanliness for endoscopic observation. During surgery, the endoscopist inserts the endoscope into the intestinal tract through the anus, and finds the lesion site of the tumor. After careful evaluation of the lesion, the boundary and scope are clarified. Next, an injection needle was used to inject an appropriate amount of normal saline or special lifting solution (such as epinephrine-normal saline mixture) into the submucosa at the bottom of the lesion to bulge the lesion tissue and separate it from the muscular layer below to form a pseudopedicle for subsequent resection. Subsequently, the snare was delivered to the lesion through the endoscopic biopsy channel, and the snare was opened and the elevated lesion tissue was accurately ensnared, ensuring that the snare was intact and there was no residual normal tissue. Then, the high-frequency electrotome was started, and current was conducted through the snare to completely remove the diseased tissue. After resection, the wound surface may suffer from hemorrhage. In this case, hot biopsy forceps can be used to coagulate and stop bleeding at the bleeding point, or argon ion coagulation (APC) can be used to stop bleeding at the wound surface to solidify and stop bleeding, thereby reducing the risk of postoperative hemorrhage. Endoscopic mucosal resection (EMR) is mainly applied to patients with early colorectal cancer who have small lesions (usually less than 2cm in diameter), bulge type or flat type, and no significant ulcer formation and submucosal fibrosis. The operation of this method is relatively simple and the damage to the intestinal tract is small.

#### 2.3.2 Endoscopic submucosal dissection

Similarly, surgery was performed on the basis of complete intestinal preparation. After the endoscope was inserted into the intestinal tract to reach the lesion, special marking liquid (such as indigo carmine solution) was used to accurately mark the edge of the lesion to determine the resection range. Then, special endoscopic cutters such as needle knife and IT knife were used to make a circular incision along the mucosal layer outside the mark point. The depth should be controlled in the submucosal layer to avoid damage to the muscular layer. After the circular incision was completed, the submucosal stripping operation was started, and the diseased tissue was gradually and completely separated from the surface of the muscular layer by using the cutter. During the process, the wound surface was constantly rinsed with normal saline to keep a clear field of view for accurate operation, and possible bleeding points were found in time and hemostatic treatment was performed. For some small vascular bleeding, coagulation can be used to stop bleeding. For larger vessels, it may be necessary to use a hemostatic clip to stop bleeding. After the dissection was completed, a comprehensive examination of the wound surface was performed again to ensure that there were no residual diseased tissues and no obvious bleeding points. Endoscopic submucosal dissection (ESD) is suitable for patients with large (diameter greater than 2cm), flat or concave type and a wide range of lesions, irregular shape and it is difficult to complete the resection of early colorectal cancer by traditional EMR. Although the operation is relatively difficult, it can completely remove a large area of diseased tissue at one time to reduce the risk of residual lesions and recurrence.

### 2.4 Intestinal flora detection method

Fresh stool samples from patients are collected, usually in specific sterile collection containers, and sent for inspection as soon as possible. To ensure the accuracy of the samples, patients should follow specific dietary restrictions and defecation specifications before collection, and avoid the recent use of drugs that may affect the intestinal flora, such as antibiotics. The DNA of microorganisms was extracted from fecal samples using the QIAamp Fast DNA Stool Mini Kit (Qiagen, Germany) following the manufacturer’s protocol (including lysis, centrifugation purification, and DNA integrity verification via agarose gel electrophoresis). Then, specific primers were used to amplify conserved regions such as the 16S rRNA gene of bacteria: the V3-V4 hypervariable region of bacterial 16S rRNA was amplified using primers 338F (5’-ACTCCTACGGGAGGCAGCA-3’) and 806R (5’-GGACTACHVGGGTWTCTAAT-3’) under the conditions: 95°C pre-denaturation for 3 min, 35 cycles of 95°C denaturation (30 s), 55°C annealing (30 s), and 72°C extension (45 s), with a final 72°C extension for 10 min. Paired-end sequencing was performed on the Illumina NovaSeq platform (Illumina, USA). Raw sequencing data were processed using QIIME2 (v2020.6): After quality filtering (Q-score > 20), reads were denoised, merged, and checked for chimeras with DADA2 to generate amplicon sequence variants (ASVs). Taxonomic classification was performed against the SILVA 132 reference database at 99% similarity. Through the analysis of these sequences, the species and relative abundance of the intestinal flora can be determined. The method can detect low abundance microorganisms in the sample and provide more comprehensive information on the composition of the intestinal flora.

### 2.5 Collection of clinical pathological parameters

The collection of clinical pathological parameters is mainly completed through detailed examination of patients’ medical records. First, basic information of patients is extracted from the electronic medical record system, including demographic characteristics such as age and gender, which helps to initially understand the overall situation of patients. For tumor-related parameters, carefully study the pathological report to identify the tumor site, such as the rectum and specific segment of the colon. Accurately record the tumor size, obtained from measured data from surgical records or imaging findings. Determining the degree of differentiation of the tumor, such as high differentiation, medium differentiation, low differentiation, etc., which reflects the similarity between the tumor cells and normal histiocytes. Checking for lymphovascular invasion, which is essential for judging the metastatic potential of the tumor. At the same time, attention should be paid to whether the tumor invasion depth is limited to the mucosal layer, submucosal layer, or the muscular layer has been involved, and so on. These parameters can comprehensively reflect the biological behavior and malignant degree of the tumor, and provide the key basis for subsequent model construction and prognosis evaluation. In addition, the principles of accuracy and completeness should be strictly followed in the collection process, to ensure the authenticity and reliability of each data, and to avoid affecting the effectiveness of research results due to data errors or deletions.

### 2.6 Prognostic evaluation method

We defined the primary endpoint as recurrence/metastasis within 1 year post-resection, based on established recurrence patterns (11, 12). Follow-up protocol included: (1) quarterly endoscopic surveillance evaluating the resection site mucosa and new mass formation. (2) cross-sectional imaging assessing lymph nodes/distant metastases. Good prognosis required meeting all criteria at 1 year: (a) no local recurrence (mass/stenosis), (b) absence of distant metastases (liver/lung imaging), (c) normal tumor markers, and (d) no cancer-related symptoms (hematochezia/weight loss). Cases failing any criterion were classified as poor prognosis.

### 2.7 Statistical analysis

Data analysis was conducted using SPSS 26.0 statistical software and R 4.3.1 software. And count data were expressed as the number of cases., and the *χ* ^2^ test or Fisher exact method was adopted. The measurement data with a normal distribution were expressed as mean ± standard deviation (SD), and the independent sample t-test was used. Multivariate Logistic regression analysis was employed to screen the risk factors for colorectal cancer invasion and metastasis, and *P* value < 0.05 was considered statistically significant. The research subjects were randomly divided into the training set and validation set at a 7:3 ratio using the ‘caret’ package of R software. The “rms” package in R software was used to establish the Nomogram model, and the “pROC” package was used to draw the ROC curve. To quantify potential overfitting, we conducted bootstrap validation (1000 iterations) with optimism correction. The optimism value represents the difference between model performance on original and bootstrap-corrected datasets. The calibration curve or Hosmer-Lemeshow test was used to evaluate the calibration of the model. The clinical decision curve (DCA) was drawn to test the practical application efficacy of the model. Significance testing was performed with the use of a two‐sided alpha level of 0.05.

## 3 Results

### 3.1 Baseline characteristics (training vs validation set)

Fifteen of the 56 patients (26.79%) in the training set had poor prognosis after treatment, and six of the 24 patients (25.00%) in the validation set had poor prognosis after treatment. There was no significant difference in the incidence of poor prognosis, intestinal flora, and clinical pathological parameters between the training set and the validation set (all *P* > 0.05) (**Table 1**).

**Table 1:** Comparison of baseline characteristics between the training set and the validation set.

Indicators		Training set (n=56)	Validation set (n=24)	*χ*²/*t*	*P* value
Age (years)		60.16 ± 7.78	58.63 ± 7.55	0.816	0.417
Gender	Male	31	14	0.060	0.806
	Female	25	10		
BMI (kg/m²)		23.47 ± 2.90	24.15 ± 3.64	0.808	0.425
Smoking history	Yes	22	9	0.023	0.881
	No	34	15		
Drinking history	Yes	33	18	1.878	0.171
	No	23	6		
Hypertension	Yes	16	8	0.181	0.670
	No	40	16		
Hyperglycemia	Yes	13	6	0.030	0.863
	No	43	18		
Hyperlipemia	Yes	11	5	0.015	0.903
	No	45	19		
Tumor diameter (cm)		2.35 ± 0.98	2.64 ± 1.32	0.956	0.346
Chao1 index		160.72 ± 28.77	153.65 ± 16.04	1.400	0.166
Shannon-Wiener index		2.36 ± 0.46	2.17 ± 0.79	1.131	0.267
UniFrac distance		0.24 ± 0.10	0.27 ± 0.16	0.781	0.441
Relative abundance of clostridium nucleatum (%)		6.17 ± 3.46	5.68 ± 2.34	0.746	0.458
Relative abundance of Bacteroides fragilis (%)		4.05 ± 1.63	4.64 ± 1.32	1.582	0.118
Relative abundance of bifidobacterium (%)		14.22 ± 4.31	13.67 ± 3.61	0.543	0.589
Relative abundance of lactobacillus (%)		11.03 ± 4.25	12.34 ± 3.42	1.332	0.187
Short chain fatty acid (μmol/g)		78.16 ± 16.34	73.35 ± 12.36	1.236	0.220
Deoxycholic acid (μmol/L)		5.85 ± 2.68	5.34 ± 2.12	0.838	0.405
Hydrogen sulfide (μmol/L)		4.50 ± 1.45	4.65 ± 2.04		

### 3.2 Univariate analysis

In the training set, univariate analysis showed that the poor prognosis group and the good prognosis group had statistical differences in age, tumor diameter, Chao1 index, Shannon-Wiener index, relative abundance of clostridium nucleatum, relative abundance of bacteroides fragilis, relative abundance of bifidobacterium, and relative abundance of lactobacillus (all *P* < 0.05) (**Table 2**).

**Table 2:** Comparison of intestinal flora and clinical pathological parameters between the poor prognosis group and the good prognosis group.

Indicators		Poor prognosis group (n=15)	Good prognosis group (n=41)	*χ*²/*t*	*P* value
Age (years)		63.53 ± 4.76	58.93 ± 8.33	2.572	0.014
Gender	Male	9	22	0.179	0.672
	Female	6	19		
BMI (kg/m²)		23.65 ± 3.21	23.40 ± 2.81	0.273	0.786
Smoking history	Yes	7	15	0.468	0.494
	No	8	26		
Drinking history	Yes	8	25	0.265	0.607
	No	7	16		
Hypertension	Yes	6	10	1.311	0.252
	No	9	31		
Hyperglycemia	Yes	5	8	1.177	0.278
	No	10	33		
Hyperlipemia	Yes	4	7	0.640	0.424
	No	11	34		
Tumor diameter (cm)		2.94 ± 1.12	2.13 ± 0.84	2.875	0.006
Chao1 index		148.21 ± 20.34	165.31 ± 30.21	2.024	0.048
Shannon-Wiener index		1.94 ± 0.32	2.51 ± 0.41	4.974	0.001
UniFrac distance		0.28 ± 0.11	0.22 ± 0.10	1.664	0.102
Relative abundance of clostridium nucleatum (%)		8.54 ± 2.21	5.31 ± 3.45	4.115	0.001
Relative abundance of Bacteroides fragilis (%)		5.25 ± 2.01	3.61 ± 1.24	2.984	0.008
Relative abundance of bifidobacterium (%)		11.44 ± 3.24	15.24 ± 4.23	3.139	0.003
Relative abundance of lactobacillus (%)		8.24 ± 3.01	12.04 ± 4.21	3.199	0.002
Short chain fatty acid (μmol/g)		72.21 ± 15.34	80.34 ± 16.32	1.674	0.100
Deoxycholic acid (μmol/L)		6.44 ± 3.21	5.64 ± 2.47	1.000	0.322
Hydrogen sulfide (μmol/L)		5.04 ± 2.14	4.31 ± 1.07	1.267	0.223

### 3.3 Multivariate analysis

The treatment prognosis was regarded as the dependent variable (0= poor, 1= good), and the factor with *P* < 0.05 in univariate analysis was regarded as the covariate. Further multivariate Logistic regression analysis showed that tumor diameter, Shannon-Wiener index, relative abundance of clostridium nucleatum, relative abundance of bacteroides fragilis, relative abundance of bifidobacterium, and relative abundance of lactobacillus were the independent influencing factors for poor prognosis after endoscopic therapy (all *P* < 0.05) (**Table 3**).

**Table 3:** Multivariate logistic regression analysis of prognostic influencing factors for early colorectal cancer treated by endoscopy.

Indicators	*B*	*S.e.*	*Wald*	*P*	*OR*	95%*CI*
Age	0.083	0.043	3.669	0.055	1.086	0.998-1.182
Tumor diameter	0.961	0.377	6.507	0.011	2.615	1.249-5.472
Chao1 index	-0.023	0.012	3.669	0.055	0.977	0.954-1.001
Shannon-Wiener index	-5.188	1.610	10.387	0.001	0.006	0.001-0.131
Relative abundance of clostridium nucleatum	0.342	0.121	7.915	0.005	1.407	1.109-1.785
Relative abundance of pseudomonas fragilis	0.693	0.232	8.947	0.003	2.000	1.270-3.149
Relative abundance of bifidobacterium	-0.264	0.098	7.286	0.007	0.768	0.634-0.930
Relative abundance of lactobacillus	-0295	0.104	8.004	0.005	0.745	0.607-0.913

### 3.4 Nomogram construction

**Figure 1:**
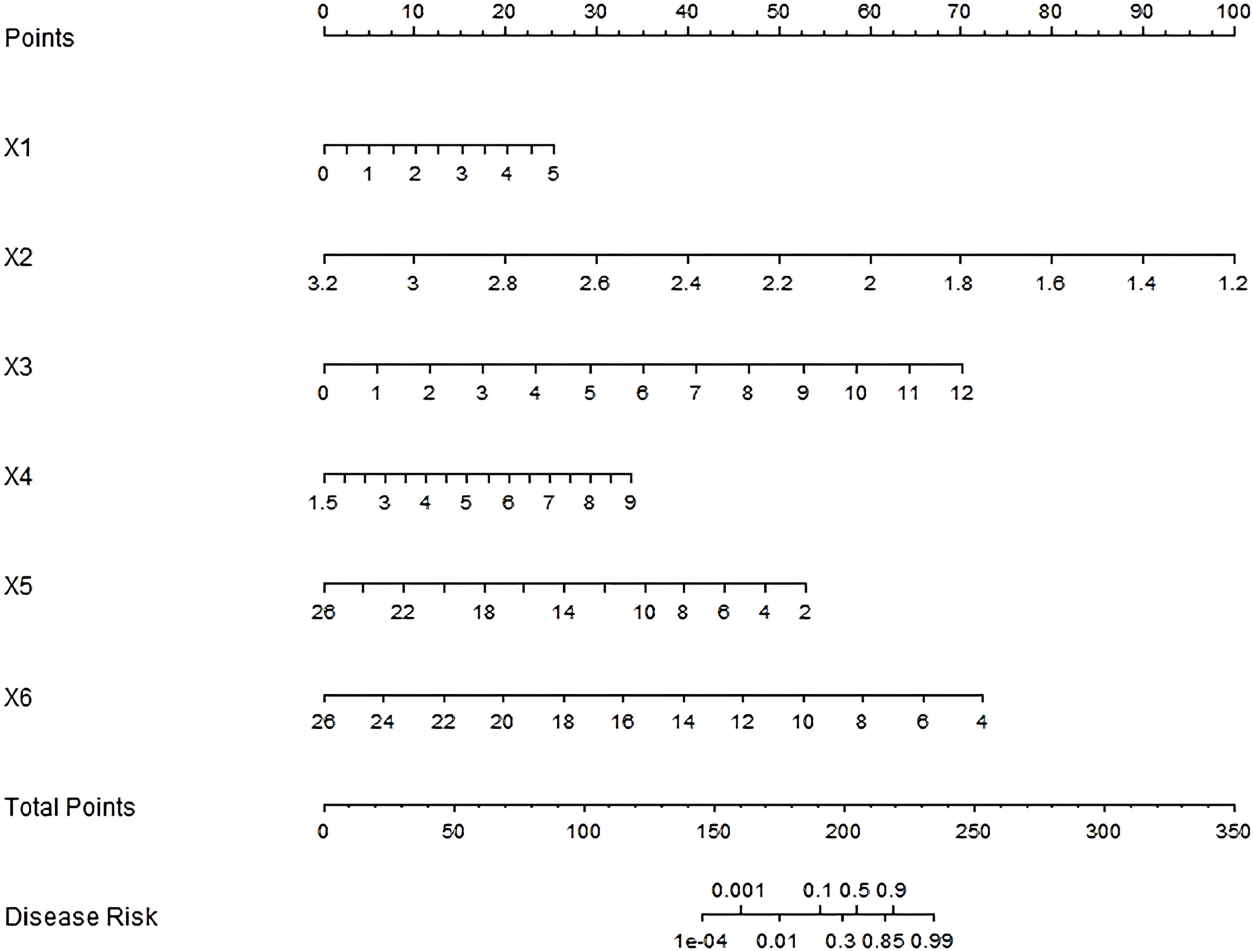
Nomogram of prognostic nomogram prediction model for endoscopic therapy. x1–x6: Tumor diameter, Shannon-Wiener index, Relative abundance of clostridium nucleatum, Relative abundance of bacteroides fragilis, Relative abundance of bifidobacterium and Relative abundance of lactobacillus.

Multicollinearity assessment confirmed all included factors demonstrated acceptable collinearity levels (VIF < 5), with a mean VIF of 2.3 (range: 1.4-4.1), supporting their independent inclusion in a nomogram prediction model. Each independent influencing factor in the model was scored, and the total score for predicting the prognosis of endoscopic treatment was calculated, which was reflected in the prediction of the incidence of poor prognosis of endoscopic treatment. A higher total score indicates a greater predicted risk of poor prognosis after endoscopic treatment (**Figure 1**).

### 3.5 Internal and external validation metrics

**Figure 2:**
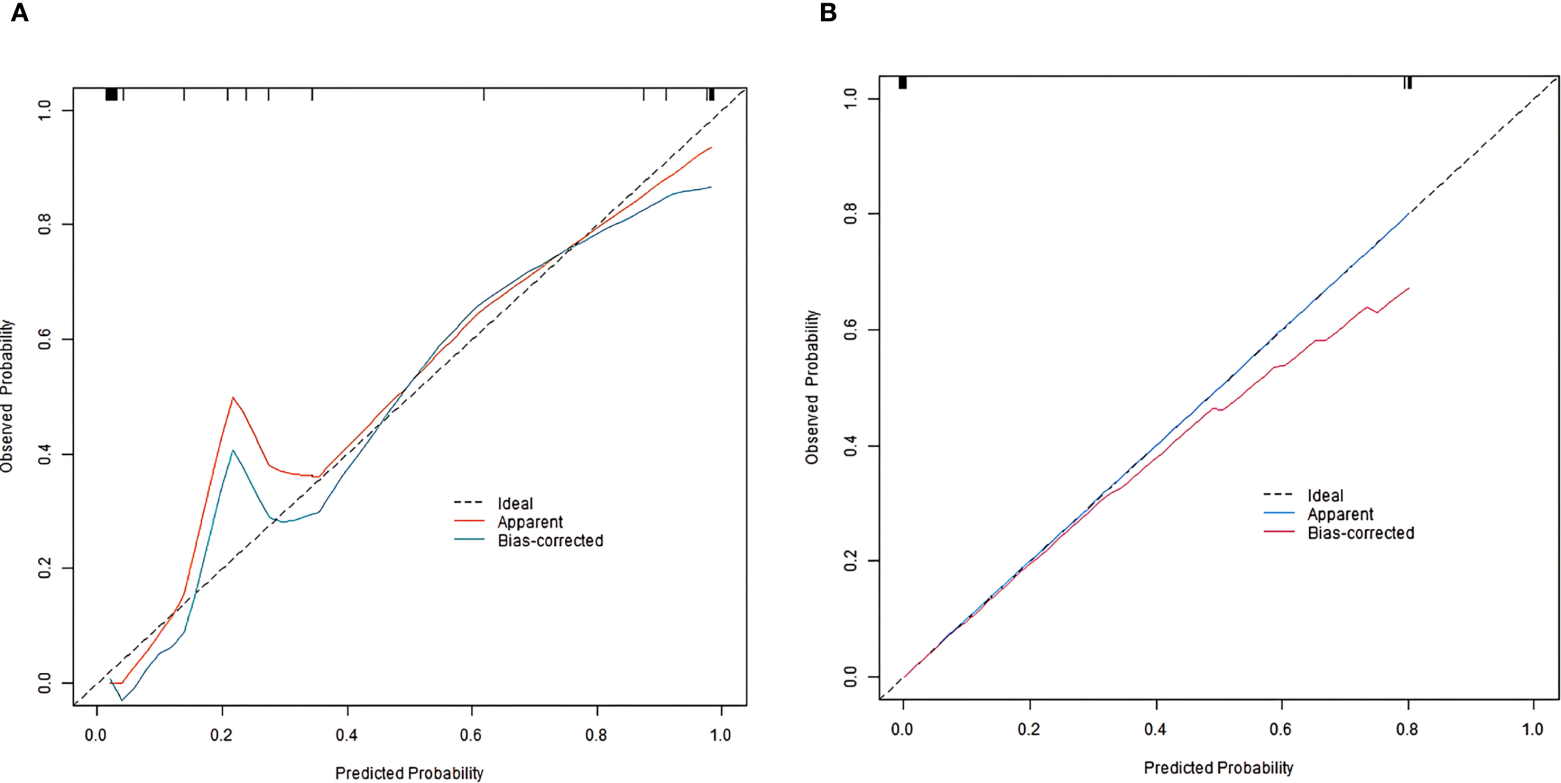
Calibration curve of prognostic prediction model for endoscopic therapy [**(A)** training set; **(B)** Validation set].

**Figure 3:**
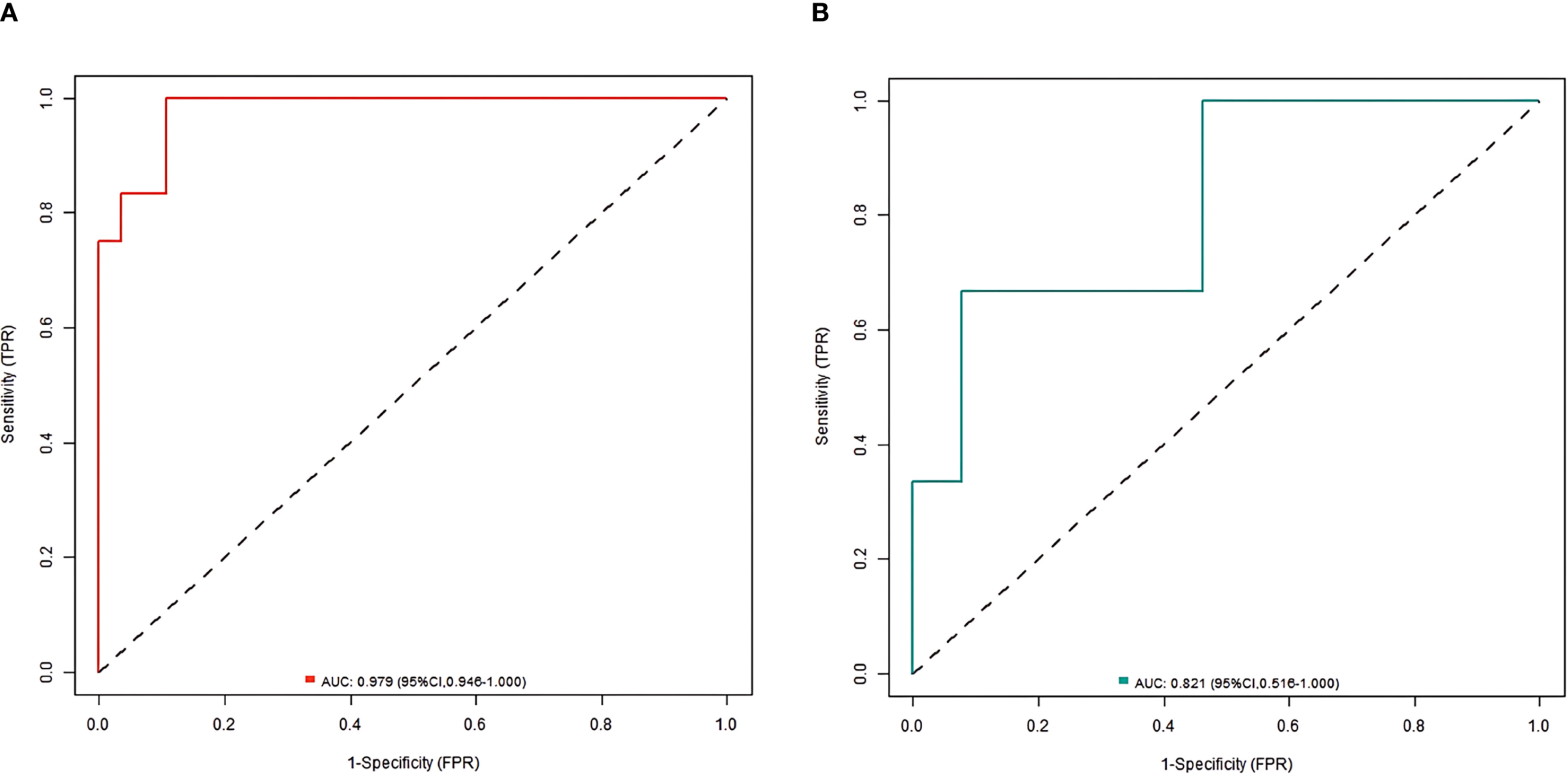
ROC curve [**(A)** training set; **(B)** Validation set].

**Figure 4:**
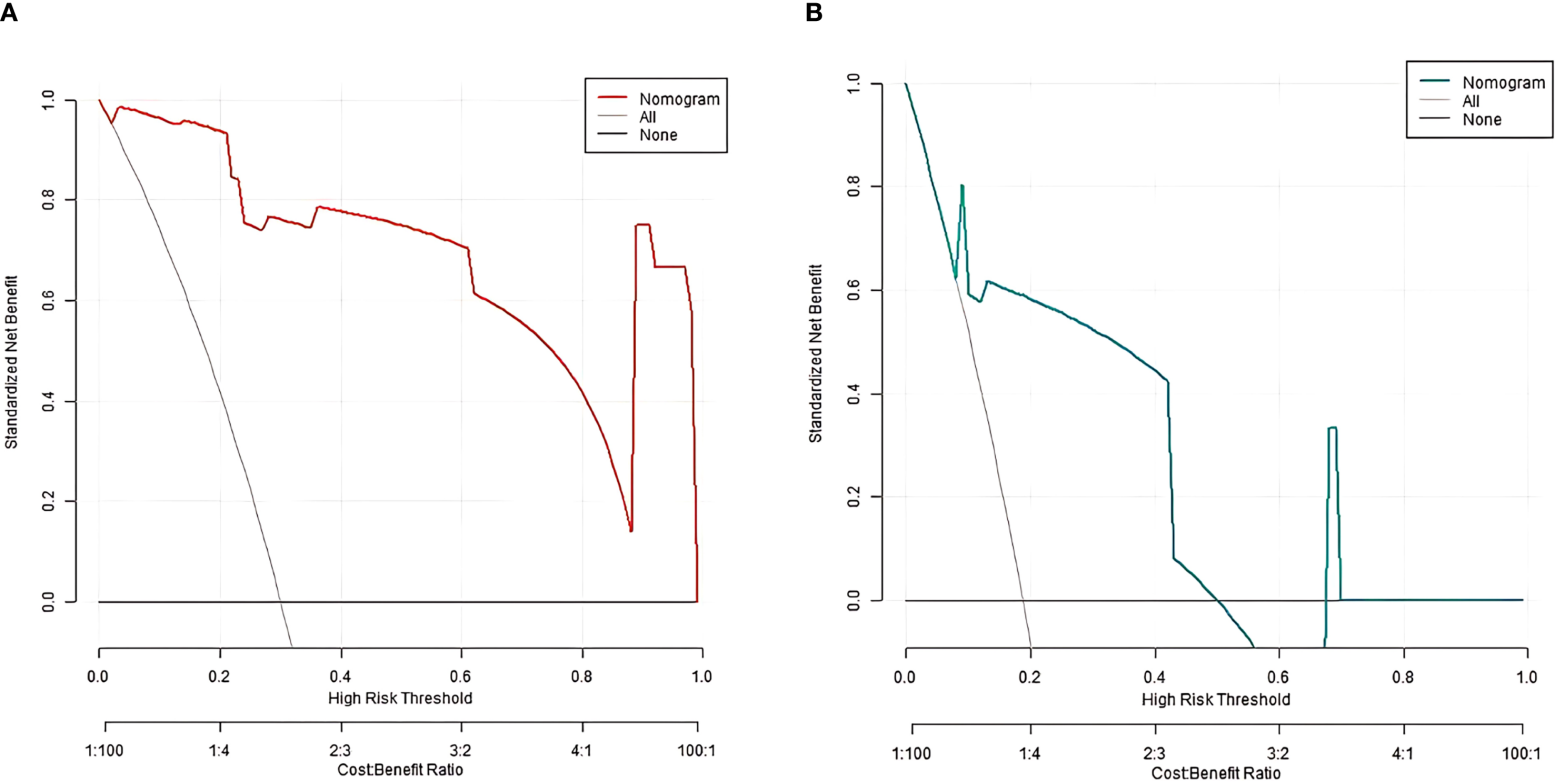
Decision curve [**(A)** training set; **(B)** Validation set].

In the training and validation sets, the nomogram model C-index was 0.989 and 0.755, respectively. After optimism correction, the model maintained good discrimination in both training (corrected C-index 0.965) and validation (corrected C-index 0.731) cohorts, suggesting reasonable stability despite the modest events-per-variable (EPV) ratio. The calibration curve showed the mean absolute errors of predicted and actual values were 0.052 and 0.129, respectively, and the Hosmer-Lemeshow test results were *χ* ^2^ = 3.289, *P* = 0.915 and *χ* ^2^ = 13.007, *P* = 0.112, respectively (**Figure 2**). The ROC curves were displayed in the training set and the validation set (**Figure 3**). The AUC of the nomogram model for predicting the prognosis of endoscopic therapy was 0.979 (95% *CI*: 0.946–1.000) and 0.821 (95% *CI*: 0.516–1.000), respectively, and the sensitivity and specificity were 0.999, 0.893, and 0.667 and 0.923, respectively. After optimism correction via bootstrap validation, the adjusted AUC was 0.968 (95% CI: 0.931–0.992) in the training set and 0.795 (95% CI: 0.503–0.947) in the validation set, indicating stable predictive performance. The decision curve shows that when the threshold probability is within the range of about 0.05–0.95, the prognostic decisions of endoscopic therapy predicted by the nomogram model constructed in this study have more clinical benefits (**Figure 4**).

## 4 Discussion

In the field of oncology medicine, the treatment strategies for early colorectal cancer have been continuously developed, and endoscopic treatment has become one of the important treatments due to its minimally invasive and effective (13–15). However, in clinical practice, even after receiving the same endoscopic treatment, there are significant differences in the prognosis of early colorectal cancer patients, which makes the accurate prediction of the prognosis of patients become the key to improve the treatment effect and the quality of life of patients (16, 17). In this study, we attempted to integrate the characteristics of intestinal flora with clinical pathological parameters and construct a nomogram model to predict the prognosis of early colorectal cancer patients after endoscopic therapy, in order to provide a more valuable reference for clinical decision-making and promote the development process of precision medicine.

In the aspect of intestinal flora, multiple flora indicators have been found to be closely related to the prognosis of endoscopic therapy. The increase in the relative abundance of clostridium nucleatum is an independent risk factor for poor prognosis, consistent with previous studies that it promotes tumor progression by activating inflammatory signaling pathways and inhibiting apoptosis (18). The increase in Bacteroides fragilis relative abundance is associated with poor prognosis, possibly due to its impact on intestinal barrier function and participation in tumor-related inflammation (19). The reduction in the relative abundance of Bifidobacterium and Lactobacilli is also important as a poor prognostic factor. Bifidobacterium and Lactobacillus are generally considered beneficial bacteria. Previous studies have reported their roles in regulating intestinal immune balance, inhibiting harmful bacteria, and participating in metabolite regulation (20). A lower Shannon-Wiener index suggests that the decrease in the diversity of the intestinal flora may lead to a decrease in the stability of the intestinal microecology, which is conducive to the survival and development of tumor cells, which also reflects the importance of the overall ecology of the intestinal flora in the prognosis of colorectal cancer (21). Additionally, tumor diameter is a key clinicopathological prognostic factor: larger tumors often have higher invasiveness and deeper infiltration, making complete endoscopic resection difficult and increasing the risk of poor prognosis (22).

While our study demonstrates the prognostic value of Fusobacterium nucleatum and associated microbial signatures, recent work by Brambilla et al. (23) highlights the complex interplay between tumor microenvironment components in colorectal cancer. Their analysis of M1/M2 macrophage polarization showed no significant correlation with survival outcomes, contrasting with the prognostic impact of metalloproteinase mutations. This divergence suggests that: (1) microbial-driven inflammation (as in our findings) and host immune responses are distinct but complementary prognostic axes; (2) future studies could simultaneously evaluate microbial colonization and immune cell profiling to elucidate potential synergies with specific immune phenotypes in influencing outcomes. The Nomogram model constructed by combining the parameters of intestinal flora with clinical pathological parameters showed good prediction efficiency. In the training set and validation set, the model performed well in C-index index, calibration curve, and area under ROC curve, indicating that the model could accurately predict the prognosis of early colorectal cancer patients after endoscopic treatment, with high reliability and stability. The nomogram’s total points (0–350) translate to disease risk probabilities (0.001–0.99), with higher scores indicating worse prognosis. For example, a patient with: Tumor diameter=3 cm (X1 = 4, 20 points), Shannon-Wiener index=2.8 (X2 = 2.2, ~50 points), Relative abundance of Clostridium nucleatum =5% (X3 = 5, ~30 points), Relative abundance of Bacteroides fragilis =6% (X4 = 6, ~20 points), Relative abundance of Bifidobacterium =10% (X5 = 10, ~35 points), Relative abundance of Lactobacilli =12% (X6 = 12, ~45 points), would sum ~200 total points, corresponding to a ~30% disease risk (interpolated from **Figure 1**). Based on the nomogram’s total score, patients were stratified into three tiers: Low-risk (<200 points, <30% risk): Routine endoscopic surveillance (every 6 months). Intermediate-risk (200–215 points, 30–85% risk): Short-interval monitoring (3 months) or prophylactic therapy. High-risk (>215 points, >85% risk): Aggressive adjuvant therapy. Our risk stratification aligns with the European Society for Medical Oncology (ESMO) guidelines (24), where high-risk thresholds trigger therapy escalation. The intermediate group (30–85% risk) may benefit from microbiome modulation, while low-risk patients reduce unnecessary interventions.

Prior to clinical adoption, three critical steps must be undertaken: (1) external validation through prospective multicenter studies involving geographically diverse populations to establish generalizability across different practice settings. (2) seamless integration with clinical decision support systems, potentially as an embedded module within existing endoscopic reporting platforms, and (3) comparative effectiveness research through pragmatic randomized trials evaluating nomogram-directed versus standard surveillance protocols. Implementation science approaches should be incorporated to identify and overcome potential adoption barriers, including clinician compliance, workflow compatibility, and health economic considerations. The advantages of this model lie in that it comprehensively considers the traditional clinical pathological factors and emerging intestinal flora factors, making up for the deficiency of single factor in the evaluation of prognosis. It can more comprehensively reflect the individual characteristics and disease state of patients, and provides an important basis for clinicians to formulate personalized treatment plan. However, this study also has some limitations. First, the sample size is relatively small, which may affect the generalization ability and accuracy of the model despite the division of the training set and the validation set. The wide CI in external validation primarily reflects cohort size constraints rather than model instability, as evidenced by internal validation metrics. Larger multicenter cohorts are needed to confirm generalizability in future. Second, the calibration of the model in the validation set needs to be improved, with a mean absolute error of 0.129, indicating a notable deviation between predicted and actual poor prognosis risks. Clinically, this could lead to misclassification of patient risk stratification: for example, a patient with an actual 20% risk of poor prognosis might be predicted to have ~7% or ~33% risk. Such errors may result in inappropriate adjustments to surveillance intensity or adjuvant therapy—overestimation could cause unnecessary medical interventions and psychological burdens, while underestimation might delay timely intervention for high-risk patients, potentially affecting treatment efficacy and long-term outcomes. Third, our study exclusively recruited patients from a single center in China, that findings may not be directly generalizable to other populations. Given known regional variations in gut microbiome composition due to diet, genetics, and environmental exposures, our findings require validation in geographically diverse cohorts. Fourth, the composition of the intestinal flora is complex and affected by a variety of factors, such as diet, lifestyle, geographical differences, etc. Among these, diet is particularly important. Our microbiota findings should be interpreted alongside dietary confounders - emulsifiers like polysorbate-80 (25): (a) selectively inhibit Bifidobacterium, and (b) enhance Bacteroides-driven inflammation via barrier disruption. This underscores the need for dietary data collection in future microbiome studies. Fifth, only a limited number of indicators related to intestinal flora and clinical pathological parameters were included in the study, which might have overlooked other potentially important factors. Further studies could explore more relevant factors to further optimize the model. In addition, our study did not employ penalized regression (e.g., LASSO or ridge regression) or automated variable selection methods. These approaches may improve robustness in high-dimensional datasets. Future studies could further improve model generalizability by incorporating the above statistical methods. Finally, the 1-year follow-up may underestimate late recurrences in high-risk subgroups; extended surveillance up to 2 years (per guidelines (12)) should be considered in future designs.

In conclusion, the Nomogram model constructed in this study still provides an important reference value and direction for the clinical research and practice of early colorectal cancer. With the in-depth study on the relationship between intestinal flora and cancer and the continuous development of big data technology, it is expected to further improve and expand this model, so that it can play a greater role in the accurate treatment and management of early colorectal cancer, and ultimately improve the prognosis and quality of life of patients, making contributions to the development of tumor personalized medicine.

## Data Availability

The raw data supporting the conclusions of this article will be made available by the authors, without undue reservation.
